# Do changes in health reveal the possibility of undiagnosed pancreatic cancer? Development of a risk-prediction model based on healthcare claims data

**DOI:** 10.1371/journal.pone.0218580

**Published:** 2019-06-25

**Authors:** Aileen Baecker, Sungjin Kim, Harvey A. Risch, Teryl K. Nuckols, Bechien U. Wu, Andrew E. Hendifar, Stephen J. Pandol, Joseph R. Pisegna, Christie Y. Jeon

**Affiliations:** 1 UCLA Fielding School of Public Health, Los Angeles, CA, United States of America; 2 Cedars-Sinai Medical Center, Los Angeles, CA, United States of America; 3 Yale School of Public Health, New Haven, CT, United States of America; 4 Kaiser Permanente Southern California, Research and Evaluation, Pasadena, CA, United States of America; 5 Veterans Affairs Greater Los Angeles Healthcare System, Los Angeles, CA, United States of America; Centro Nacional de Investigaciones Oncologicas, SPAIN

## Abstract

**Background and objective:**

Early detection methods for pancreatic cancer are lacking. We aimed to develop a prediction model for pancreatic cancer based on changes in health captured by healthcare claims data.

**Methods:**

We conducted a case-control study on 29,646 Medicare-enrolled patients aged 68 years and above with pancreatic ductal adenocarcinoma (PDAC) reported to the Surveillance Epidemiology an End Results (SEER) tumor registries program in 2004–2011 and 88,938 age and sex-matched controls. We developed a prediction model using multivariable logistic regression on Medicare claims for 16 risk factors and pre-diagnostic symptoms of PDAC present within 15 months prior to PDAC diagnosis. Claims within 3 months of PDAC diagnosis were excluded in sensitivity analyses. We evaluated the discriminatory power of the model with the area under the receiver operating curve (AUC) and performed cross-validation by bootstrapping.

**Results:**

The prediction model on all cases and controls reached AUC of 0.68. Excluding the final 3 months of claims lowered the AUC to 0.58. Among new-onset diabetes patients, the prediction model reached AUC of 0.73, which decreased to 0.63 when claims from the final 3 months were excluded. Performance measures of the prediction models was confirmed by internal validation using the bootstrap method.

**Conclusion:**

Models based on healthcare claims for clinical risk factors, symptoms and signs of pancreatic cancer are limited in classifying those who go on to diagnosis of pancreatic cancer and those who do not, especially when excluding claims that immediately precede the diagnosis of PDAC.

## Introduction

Over 50,000 new cases and 40,000 deaths from pancreatic cancer occur annually in the U.S.[1] With a 5-year survival proportion below 10%, pancreatic cancer is the deadliest solid organ cancer. [1, 2] If current trends continue, pancreatic cancer will become the second leading cause of cancer death by 2030. [3] Most pancreatic cancer patients have advanced stage disease at diagnosis; [1] therefore, strategies for detecting pancreatic cancer earlier could expand treatment options and improve survival.

Metabolic and gastrointestinal changes are strongly associated with incident pancreatic cancer. For example, people with new diagnoses of diabetes are at ≥4 -fold increased risk of pancreatic cancer diagnosis in the next two years. [4–6] In some patients, new-onset diabetes reflects a paraneoplastic phenomenon arising from tumor in the pancreas. [7, 8] Development of pancreatic ductal adenocarcinoma (PDAC) is also often marked with unintentional weight loss. [9] Recent diagnosis of pancreatitis is also strongly associated with PDAC risk with an odds ratio (OR) of 13.6, reflecting potential misdiagnosis of PDAC as pancreatitis, or the causation of pancreatitis by the developing neoplasm. [10] Similarly, recent initiation of proton-pump inhibitor (PPI) use is related to PDAC risk (OR = 6.2), suggesting that PDAC-related abdominal discomfort is sometimes treated as dyspepsia. [11]

Collectively, changes in health as manifested in healthcare claims could potentially be used to detect PDAC at earlier stages. Previous prediction models for PDAC that have incorporated data on changes in health have shown modest discriminative power, but have varied applicability to the general population in the U.S. [11–13] We hypothesize that predictive modeling using healthcare claims from a national insurance program in the U.S. can help identify older adults who are at high risk of pancreatic cancer. Using Medicare-linked data on cancer diagnoses reported to Surveillance, Epidemiology, and End Results (SEER) cancer registries between January 2004 and December 2011, we conducted a matched retrospective case-control study to develop a prediction model for pancreatic cancer.

## Materials and methods

### Data sources

The SEER database includes information on cancer incidence and survival from population-based registries in geographic regions currently comprising approximately 28% of the U.S. population. [14] Linkage of SEER to Medicare claims on inpatient and outpatient procedures and diagnoses offers unique population-based source of information on patterns of care before and after diagnosis that can be used for epidemiological and health services research. [15, 16] For the purposes of the current analyses, we extracted pathology and diagnosis information on PDAC cases from SEER, selected controls from a matched random sample of Medicare members, and extracted covariate data from Medicare claims. SEER-Medicare data pertaining to pancreatic cancer cases and controls were obtained and analyzed as a limited data set without direct identifiers. The Institutional Review Board of Cedars-Sinai Medical Center has approved this study.

### Selection of cases

Based on topography code C25.x and ICD-O-3 histology codes for adenocarcinoma of the pancreas (8000, 8010, 8020, 8021, 8022, 8050, 8140, 8141, 8211, 8230, 8260, 8441, 8450, 8453, 8470, 8471, 8472, 8473, 8480, 8481, 8500, 8503, 8521), [17] we identified all newly diagnosed PDAC patients at least 68 years old. We chose 68 years as the minimum age so that eligible patients had at least three years enrollment duration in Medicare Parts A and B prior to diagnosis of pancreatic cancer. We only included people with PDAC that was confirmed by microscopy, laboratory test, direct visualization, or imaging, and excluded cases with unknown months of diagnoses or those diagnosed at autopsy. Because SEER reports only the month and year of cancer diagnosis, we set the 1^st^ of the month as the diagnosis date for the purposes of designating pre-diagnosis claims.

### Selection of controls

Using the 5% random sample of Medicare beneficiaries, we selected 3 controls for each case and matched them by sex, 5-year age group and year of diagnosis. Controls were free of pancreatic cancer as of July 1^st^ of the same year as case diagnosis, and had been enrolled in Medicare A and B for at least three years as of that point in time. This methodology parallels control selection methods by Engels, et al. [18] The same control was allowed to be sampled across multiple years; however each control was only sampled once in a calendar year. Index date was defined as July 1^st^ of the same year as the matched case.

### Covariates

On the basis of consensus between investigators with expertise in oncology, gastroenterology and epidemiology and published literature, we selected clinical health changes known to be associated with PDAC, including acute pancreatitis, chronic pancreatitis, any abdominal pain, chest pain, diabetes mellitus, weight loss/anorexia/cachexia, nausea and/or vomiting, digestive problems, dyspepsia/gastritis/peptic ulcer disease, fatigue, itching/pruritis, depression, jaundice, gallbladder disease, acute cholecystitis, and esophageal reflux. S1 Table lists these covariates and their corresponding ICD-9 codes. We extracted ICD-9 coded claims for these factors from Medicare inpatient and outpatient data files.

### Healthcare access

Healthcare claims are more likely to be consistent among patients who make use of recommended preventive services. A proxy indicator for such individuals among Medicare enrollees is compliance with the annual influenza vaccine recommendation, which is correlated with health literacy and motivation to seek care. [19, 20] To adjust for healthcare access, we included influenza vaccination in all models. Compliance with the vaccine recommendation was determined by extracting claims data on receipt of influenza vaccination (HCPCS codes G0008, Q2035, Q2036, Q2037, Q2038) in the 12-month period prior to index date.

### Statistical analysis plan

To visualize the trends of claims for covariates of interest prior to diagnosis with PDAC and to identify a pre-diagnosis window of time when such trends diverge between cases and controls, we summarized the ratios of percent of cases to controls who had healthcare claims for the covariates of interest within 24 months prior to diagnosis. The 24-month history was divided into 3-month intervals (total of 8 quarter years). For the purpose of the main prediction model, we included claims within 15 months prior to PDAC diagnosis or index date to incorporate as many covariates that diverge between the cases and the controls, as well as to have sufficient lead time prior to pancreatic cancer diagnosis to identify potentially useful early detection signals.

To describe covariate distributions of the case and control sample groups, we computed frequencies and percentages for categorical variables and medians and interquartile ranges for continuous variables. The primary outcome was the occurrence of PDAC. We compared covariate distributions between the case and control groups by Wilcoxon rank-sum statistics or chi-square statistics, as appropriate. To quantify associations between the covariates and the outcome, we constructed unconditional logistic regression models under adjustment for the matching variables: sex, age group, and year of diagnosis. Because we sampled some patients more than once, we accounted for repeated measurements on the same control across multiple years by robust variance estimates. Variables initially considered for inclusion in the multivariable model included race and influenza vaccine status and all of the covariates described above.

Model selection was conducted by stepwise variable selection procedure based on Quasi-likelihood under the Independence model Criterion (QIC) statistic. [21, 22] The final multivariable model was chosen by the lowest QIC value, a statistical alternative to Akaike’s information criterion [23] but for correlated data. Age group, sex, year of diagnosis, race and influenza vaccine status were kept in the model regardless of statistical significance.

### Model performance

We evaluated the sensitivity of the models at specificities of 99% or higher, 95–99%, and <95%. We set thresholds based on specificity, rather than sensitivity, given the infrequency of the disease, and the high cost of false positivity (e.g., patient anxiety, costly imaging). Performance of the models on predicting occurrence of pancreatic cancer was further assessed with measures of discrimination and calibration. [24] Discrimination was evaluated by receiver operating characteristic (ROC) curve and area under the ROC curve (AUC, or C-index).^16^ Calibration of the prediction models was evaluated with calibration slope intercepts, and graphically assessed with predicted versus observed probability of the occurrence of PDAC based on the loess algorithm. [25] Internal validation of the models was performed by estimating and correcting for possible overfitting and optimism in the model performance estimates by bootstrap methods with 1000 replicates. [25–27]

### Sensitivity analyses

To evaluate how the prediction model may have been influenced by claims immediately preceding the diagnosis of PDAC, which may reflect diagnostic work-up for cancer, we conducted sensitivity analyses excluding claims occurring less than 3 months prior to PDAC diagnosis. Because new-onset diabetes can be an early indicator of pancreatic cancer, [7, 8] and has been the focus of published prediction models, [11–13] we also performed sensitivity analyses among those with new claims for diabetes within 15 months prior to the index date, without any claim for diabetes prior to this period. Finally, a separate prediction model was also created based on claims presented 16–24 months prior to the index date, to evaluate possible prediction utility further before diagnosis. To consider the influence of including weak associations in the prediction models, we also constructed models with parsimonious selection of variables that were associated with PDAC with OR > 2 for each of the models above. In all models, except for new-onset diabetes, we included relevant claims within the specified time period whether or not they were the first ever claim for the condition.

All statistical analyses were performed using SAS 9.4 (SAS Institute, Inc., Cary, North Carolina) and R package version 3.5.0 (The R Foundation for Statistical Computing). The Institutional Review Board of Cedars-Sinai Medical Center approved the study. We followed the STROBE guidelines for reporting of results of case-control studies, [28] and the PROBAST guidelines for reporting on potential bias and applicability of prediction models. [29]

## Results

In total, 51,540 non-deceased pancreatic cancer patients with known diagnosis month and year were reported to SEER between 2004 and 2011; 44,882 of these were malignant primary PDAC. Diagnosis was confirmed by microscope or laboratory tests or by imaging in 41,305 cases, of whom 29,646 met all our study eligibility criteria. Of note, 23,332 of the cases were microscopically confirmed (79%). We selected 88,938 controls matched to the cases. Table 1 provides characteristics of the cases and controls.

**Table 1:** Patient characteristics and presence of healthcare claims for covariates prior to pancreatic ductal adenocarcinoma diagnosis. Data are presented as number of patients (%).

Variable	Pancreatic cancer (N = 29646)	Pancreatic cancer-Free (N = 88938)	P-value
Sex			
Male	12719 (42.9)	38157 (42.9)	1.000
Female	16927 (57.1)	50781 (57.1)	
Age at diagnosis (Years)			
68–70	3648 (12.3)	10789 (12.1)	0.003
71–75	7108 (24.0)	20880 (23.5)	
76–80	7519 (25.4)	22457 (25.3)	
81–85	6289 (21.2)	18674 (21.0)	
86+	5082 (17.1)	16138 (18.2)	
Race			
Black	2974 (10.0)	6813 (7.7)	< .001
Other	2592 (8.7)	8402 (9.5)	
White	24080 (81.2)	73723 (82.9)	
Influenza vaccination in the last 12 months			
Yes	11201 (37.78)	32637 (36.7)	< .001
***Presence of healthcare claims for clinical diagnoses***			
Acute pancreatitis			
≤ 15 months of index date	2045 (6.90)	483 (0.54)	< .001
Excluding final 3 months	721 (2.43)	389 (0.44)	< .001
Chronic pancreatitis			
≤ 15 months of index date	807 (2.72)	135 (0.15)	< .001
Excluding final 3 months	344 (1.16)	112 (0.13)	< .001
Diabetes mellitus			
≤ 15 months of index date	10611 (35.8)	20414 (23.0)	< .001
Excluding final 3 months	9320 (31.4)	19034 (21.4)	< .001
Dyspepsia, gastritis, peptic ulcer disease			
≤ 15 months of index date	1697 (5.72)	2373 (2.67)	< .001
Excluding final 3 months	881 (2.97)	1960 (2.20)	< .001
Gallbladder disease			
≤ 15 months of index date	249 (0.84)	137 (0.15)	< .001
Excluding final 3 months	83 (0.28)	115 (0.13)	< .001
Acute cholecystitis			
≤ 15 months of index date	286 (0.96)	218 (0.25)	< .001
Excluding final 3 months	122 (0.41)	176 (0.20)	< .001
Depression			
≤ 15 months of index date	2493 (8.41)	7025 (7.90)	0.005
Excluding final 3 months	1918 (6.47)	6029 (6.78)	0.065
***Presence of healthcare claims for symptoms and signs***			
Any abdominal pain			
≤ 15 months of index date	11218 (37.8)	13423 (15.1)	< .001
Excluding final 3 months	5389 (18.2)	11255 (12.7)	< .001
Chest pain			
≤ 15 months of index date	8359 (28.2)	18868 (21.2)	< .001
Excluding final 3 months	6178 (20.8)	16044 (18.0)	< .001
Gastrointestinal symptoms			
≤ 15 months of index date	4302 (14.5)	7134 (8.02)	< .001
Excluding final 3 months	2420 (8.16)	5793 (6.51)	< .001
Esophageal reflux			
≤ 15 months of index date	5277 (17.8)	11245 (12.6)	< .001
Excluding final 3 months	3551 (12.0)	9591 (10.8)	< .001
Jaundice			
≤ 15 months of index date	2531 (8.54)	196 (0.22)	< .001
Excluding final 3 months	328 (1.11)	162 (0.18)	< .001
Weight loss / Anorexia / Cachexia			
≤ 15 months of index date	4956 (16.7)	4075 (4.58)	< .001
Excluding final 3 months	1962 (6.62)	3263 (3.67)	< .001
Nausea and/or vomiting			
≤ 15 months of index date	3610 (12.2)	5378 (6.05)	< .001
Excluding final 3 months	1820 (6.14)	4350 (4.89)	< .001
Malaise/Fatigue			
≤ 15 months of index date	46 (0.16)	89 (0.10)	0.015
Excluding final 3 months	35 (0.12)	87 (0.10)	0.347
Itching/pruritis			
≤ 15 months of index date	678 (2.29)	1092 (1.23)	< .001
Excluding final 3 months	356 (1.20)	896 (1.01)	0.005

### Pre-diagnostic claims history in cases and controls

**Fig 1:**
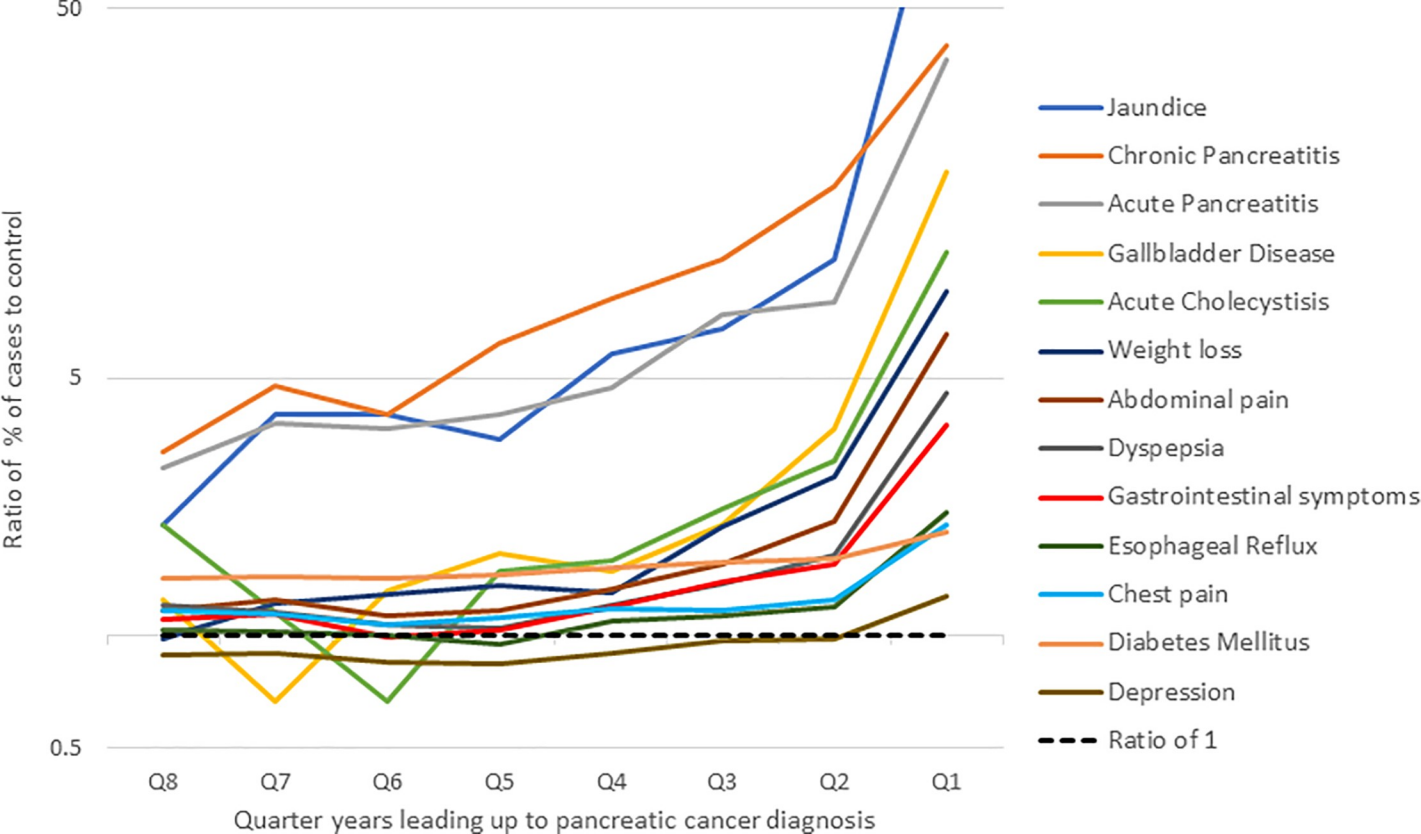
Ratio of percentage of cases to controls with a healthcare claim for covariates of pancreatic cancer within 24-months prior to pancreatic cancer diagnosis, by 3-month intervals.

Fig 1 illustrates the relative proportion of claims for each indicator in PDAC cases vs. control within 24 months prior to the index date. Covariates such as chronic pancreatitis, acute pancreatitis, jaundice and poorly controlled diabetes are present in greater frequency in cases vs. control from as early as 24 months prior to cancer diagnosis or matched date. In addition to these factors, covariates such as upper abdominal pain, gallbladder disease, digestive symptoms and weight loss were present in greater proportion of patients with pancreatic cancer than in controls within 15 months prior to cancer diagnosis or matched date. All factors were more elevated in cases vs. controls in the last 3 months prior to cancer diagnosis, and ratios for cases vs. controls steeply increased in this quarter. (Fig 1) A summary of proportions of cases and controls with claims for each covariate by quarter is provided in S2 Table.

### Multivariable results

Table 2 shows the results of multivariable analyses. In the analyses focusing on the 15 months before diagnosis, factors significantly associated with PDAC included black race (OR = 1.14) relative to white race, and presence of at least 1 claim for acute pancreatitis, (OR = 4.72), chronic pancreatitis (OR = 3.72), diabetes mellitus (OR = 1.52), dyspepsia (OR = 1.25), gallbladder disease (OR = 1.34), any abdominal pain (OR = 2.38), weight loss (OR = 2.70), and jaundice (OR = 24.0). Influenza vaccination (OR = 0.82), depression (OR = 0.72), and chest pain (OR = 0.89) were significantly associated with reduced PDAC risk.

**Table 2:** Multivariable analysis of incidence of pancreatic cancer by covariates present at healthcare visits within 15 months prior to diagnosis of pancreatic cancer or matched date in controls.

Variable	Multivariable model		Multivariable model excluding last 3 months of claims	
	Odds Ratio (95% CI)	P-value	Odds Ratio (95% CI)	P-value
Race *				
Black	1.14 (1.08–1.20)	< .001	1.22 (1.16–1.28)	< .001
Other	0.88 (0.83–0.93)	< .001	0.90 (0.95–0.94)	< .001
Influenza vaccination in the last 12 months	0.82 (0.80–0.85)	< .001	0.96 (0.94–0.99)	0.006
***Presence of healthcare claims for clinical diagnoses***				
Acute pancreatitis	4.72 (4.20–5.30)	< .001	3.11 (2.71–3.57)	< .001
Chronic pancreatitis	3.72 (2.98–4.64)	< .001	3.48 (2.74–4.41)	< .001
Diabetes mellitus	1.52 (1.47–1.57)	< .001	1.60 (1.55–1.65)	< .001
Dyspepsia, gastritis, peptic ulcer disease	1.25 (1.16–1.34)	< .001	1.08 (0.99–1.18)	0.067
Gallbladder disease	1.34 (1.02–1.76)	0.037	†	
Depression	0.72 (0.68–0.76)	< .001	0.80 (0.76–0.85)	< .001
***Presence of healthcare claims for symptoms and signs***				
Any abdominal pain	2.38 (2.30–2.47)	< .001	1.26 (1.21–1.31)	< .001
Chest pain	0.89 (0.86–0.92)	< .001	†	
Esophageal reflux	0.94 (0.90–0.99)	0.008	†	
Weight loss	2.70 (2.57–2.84)	< .001	1.57 (1.48–1.67)	< .001
Jaundice	24.01 (20.63–27.95)	< .001	3.77 (3.10–4.57)	< .001
Nausea and/or vomiting	†		0.90 (0.84–0.96)	< .001

118584 observations were used in the multivariable models, which are adjusted for sex, age at diagnosis, and year of diagnosis, and takes into account correlated data from the same subject.

* White race as a reference level.

† Dropped out of the multivariable model.

Excluding claims from the final 3 months before index date weakened these associations. For example, acute pancreatitis and jaundice were associated with 3.1-fold and 3.8-fold increased risk of PDAC. The strength of the association for diabetes did not change with the exclusion of the final 3 months of claims, but that for weight loss decreased from OR of 2.70 to 1.57. Dyspepsia and gallbladder disease were associated in the 1–15 month model were no longer significantly associated with PDAC risk when we excluded claims from the final 3 months.

Table 3 presents the covariate distributions between the case and control groups among those with new-onset diabetes, comprising 7.8% of the cases (n = 2,319), and 3.8% of the controls (n = 3,400). The results of the multivariable model for persons with new-onset diabetes are presented in Table 4 and show similar trends to the entire case-control sample. Patients with acute pancreatitis, chronic pancreatitis, abdominal pain, weight loss, and jaundice experienced increased risk of PDAC. Also of note, in persons with new claims for diabetes, poorly controlled diabetes was additionally associated with PDAC risk. As in the model based on the full subject sample, depression was negatively associated with PDAC risk. Excluding the final 3 months of claims eligibility attenuated the associations between the covariates and PDAC risk. Regardless, acute pancreatitis, chronic pancreatitis, abdominal pain, weight loss, and jaundice were associated with PDAC risk. Poorly controlled diabetes and nausea/vomiting were no longer associated with PDAC risk and omitted from the model, while depression and chest pain were inversely associated with PDAC risk.

**Table 3:** Baseline patient characteristics stratified by incidence of pancreatic cancer in persons with new-onset DM. Data are presented as number of patients (%).

Variable	Pancreatic cancer (N = 2319)	Pancreatic cancer-Free (N = 3400)	P-value
Sex			
Male	1000 (43.12)	1517 (44.62)	0.263
Female	1319 (56.88)	1883 (55.38)	
Age at diagnosis (Years)			
68–70	341 (14.7)	466 (13.71)	0.001
71–75	583 (25.14)	801 (23.56)	
76–80	622 (26.82)	818 (24.06)	
81–85	456 (19.66)	745 (21.91)	
86+	317 (13.67)	570 (16.76)	
Race			
Black	249 (10.74)	315 (9.26)	0.031
Other	201 (8.67)	351 (10.32)	
White	1869 (80.6)	2734 (80.41)	
Influenza vaccination in the last 12 months			
Yes	968 (41.74)	1434 (42.18)	0.744
No	1351 (58.26)	1966 (57.82)	
***Presence of healthcare claims for clinical diagnoses***			
Acute pancreatitis			
≤ 15 months of index date	208 (8.97)	43 (1.26)	< .001
Excluding final 3 months	85 (3.67)	33 (0.97)	< .001
Chronic pancreatitis			
≤ 15 months of index date	81 (3.49)	13 (0.38)	< .001
Excluding final 3 months	36 (1.55)	11 (0.32)	< .001
Poorly controlled diabetes mellitus			
≤ 15 months of index date	807 (34.8)	859 (25.26)	< .001
Excluding final 3 months	497 (21.43)	663 (19.5)	0.074
Dyspepsia, gastritis, peptic ulcer disease			
≤ 15 months of index date	176 (7.59)	138 (4.06)	< .001
Excluding final 3 months	86 (3.71)	109 (3.21)	0.304
Gallbladder disease			
≤ 15 months of index date	25 (1.08)	14 (0.41)	0.003
Excluding final 3 months	12 (0.52)	11 (0.32)	0.255
Acute cholecystitis			
≤ 15 months of index date	33 (1.42)	17 (0.5)	< .001
Excluding final 3 months	16 (0.69)	14 (0.41)	0.153
Depression			
≤ 15 months of index date	229 (9.87)	443 (13.03)	< .001
Excluding final 3 months	169 (7.29)	365 (10.74)	< .001
***Presence of healthcare claims for symptoms and signs***			
Any abdominal pain			
≤ 15 months of index date	1128 (48.64)	774 (22.76)	< .001
Excluding final 3 months	541 (23.33)	657 (19.32)	< .001
Chest pain			
≤ 15 months of index date	824 (35.53)	1188 (34.94)	0.646
Excluding final 3 months	575 (24.8)	1014 (29.82)	< .001
Gastrointestinal symptoms			
≤ 15 months of index date	410 (17.68)	414 (12.18)	< .001
Excluding final 3 months	223 (9.62)	344 (10.12)	0.533
Esophageal reflux			
≤ 15 months of index date	522 (22.51)	618 (18.18)	< .001
Excluding final 3 months	358 (15.44)	514 (15.12)	0.741
Jaundice			
≤ 15 months of index date	283 (12.2)	18 (0.53)	< .001
Excluding final 3 months	49 (2.11)	13 (0.38)	< .001
Cachexia			
≤ 15 months of index date	597 (25.74)	249 (7.32)	< .001
Excluding final 3 months	219 (9.44)	188 (5.53)	< .001
Nausea and/or vomiting			
≤ 15 months of index date	325 (14.01)	339 (9.97)	< .001
Excluding final 3 months	165 (7.12)	276 (8.12)	0.163
Fatigue			
≤ 15 months of index date	^	^	
Excluding final 3 months	^	^	
Itching/pruritis			
≤ 15 months of index date	75 (3.23)	73 (2.15)	0.011
Excluding final 3 months	33 (1.42)	56 (1.65)	0.502

^ Statistic not presented per SEER guidelines as cell counts were less than 11.

**Table 4:** Multivariable analysis of incidence of pancreatic cancer *among persons with new-onset diabetes* by covariates present at healthcare visits within 15 months prior to diagnosis of pancreatic cancer or matched date in controls.

Variable	Multivariable model		Multivariable model excluding last 3 months of claims	
	Odds Ratio (95% CI)	P-value	Odds Ratio (95% CI)	P-value
Race *				
Black	0.99 (0.81–1.20)	0.889	1.12 (0.93–1.35)	0.217
Other	0.75 (0.61–0.92)	0.005	0.78 (0.65–0.95)	0.012
Influenza vaccination in the last 12 months	0.92 (0.81–1.03)	0.158	1.01 (0.91–1.14)	0.800
***Presence of healthcare claims for clinical diagnoses***				
Acute pancreatitis	3.89 (2.67–5.68)	< .001	2.89 (1.82–4.58)	< .001
Chronic pancreatitis	2.42 (1.23–4.76)	0.010	1.92 (0.91–4.04)	0.087
Poorly controlled diabetes mellitus	1.63 (1.43–1.85)	< .001	†	
Depression	0.49 (0.39–0.61)	< .001	0.64 (0.52–0.79)	< .001
***Presence of healthcare claims for symptoms and signs***				
Any abdominal pain	2.52 (2.21–2.88)	< .001	1.25 (1.08–1.44)	0.002
Chest pain	†		0.76 (0.66–0.86)	< .001
Weight loss / Anorexia Cachexia	3.64 (3.06–4.33)	< .001	1.82 (1.47–2.25)	< .001
Jaundice	17.95 (10.93–29.48)	< .001	3.68 (1.94–7.00)	< .001
Nausea and/or vomiting	0.74 (0.60–0.91)	0.005	†	

5719 observations were used in the multivariable models, which are adjusted for sex, age at diagnosis, and year of diagnosis, and takes into account correlated data from the same subject.

* White race as a reference level.

† Dropped out of the multivariable model.

### Model performance

Table 5 presents the performance measures of the multivariable regression models. The AUC for the prediction model based on claims 1–15 months prior to PDAC, was 0.683. Excluding claim from 3 months prior to index date reduced the AUC to 0.578. In contrast, excluding non-microscopically confirmed cases increased the AUC slightly to 0.703. Optimism-corrected AUCs confirmed the performance measures. We found good calibration between the development and validation models with the optimism-corrected slope and intercept of 0.996 and -0.004, respectively, for the 1–15 months prediction model, and of 0.988 and -0.012 for the model excluding 3 months of claims. At a specificity of 99%, the prediction model based on ≤15 months claims yielded sensitivity of 16.2%, and the model excluding 3 months of claims yielded sensitivity of 4.7%. A sensitivity of 16.2% translates to 1-year positive predictive value of 1.2% if applied to a population aged ≥65 in whom the annual risk of PDAC is 70 cases per 100,000.[30] A sensitivity of 4.7% translates to 1-year positive predictive value of 0.33% if applied to the same population.

**Table 5:** Performance characteristics for prediction models of pancreatic cancer based on healthcare claims.

Population	Time period for claims included in the multivariable model	AUC (95% CI)	Optimism-corrected AUC (95% CI)	Specificity	Sensitivity
All cases and controls (cases 29,646; controls 88,938)	A. ≤15 months period prior to index date	0.683 (0.680–0.687)	0.682 (0.678–0.686)	≥ 99%	< 16.17%
				95–98.9%	16.17–27.84%
				< 95%	> 27.84%
All cases and controls (cases 29,646; controls 88,938)	Model A excluding last 3 months of claims prior to index date	0.578 (0.575–0.582)	0.577 (0.573–0.581)	≥ 99%	< 4.71%
				95–98.9%	4.71–11.79%
				< 95%	> 11.79%
Cases and controls (cases 23,332; controls 88,938)	Model A excluding non-microscopically confirmed cases	0.703 (0.699–0.707)	0.702 (0.698–0.706)	≥ 99%	< 18.14%
				95–98.9%	18.14–30.05%
				< 95%	> 30.05%
Cases and controls with new-onset diabetes (cases 2,319; controls 3,400)	B. ≤15 months period prior to index date	0.735 (0.721–0.748)	0.730 (0.717–0.744)	≥ 99%	< 18.24%
				95–98.9%	18.24–33.89%
				< 95%	> 33.89%
Cases and controls with new-onset diabetes (cases 2,319; controls 3,400)	Model B excluding last 3 months of claims prior to index date	0.635 (0.621–0.650)	0.626 (0.612–0.641)	≥ 99%	< 4.44%
				95–98.9%	4.44–13.76%
				< 95%	> 13.76%
Cases and controls with new-onset diabetes (cases 1,873; controls 3,400)	Model B excluding non-microscopically confirmed cases	0.754 (0.739–0.768)	0.747 (0.733–0.761)	≥ 99%	< 20.12%
				95–98.9%	20.12–36.15%
				< 95%	> 36.15%

The AUC and optimism-corrected AUC of the prediction model in persons with new-onset diabetes reached 0.735 and 0.730 for all claims within 15 months of the index ate, 0.635 and 0.626 excluding claims from the final 3 months, and 0.754 and 0.747 excluding cases not confirmed microscopically, respectively. Good calibrations remained for the prediction models in persons with new-onset diabetes. For these subjects, at a specificity of 99%, the prediction model on ≤15 months claims yielded sensitivity of 18.2%, and excluding 3 months of claims, 4.4%. The corresponding 1-year positive predictive values were 3.5% and 0.87%, respectively, assuming baseline annual risk of PDAC of 200 cases per 100,000 person-years after new-onset diabetes.[31]

For each of the models presented above, we also examined parsimonious models including only risk factors associated with PDAC with OR > 2 (acute pancreatitis, chronic pancreatitis, diabetes, abdominal pain, weight loss and jaundice). Parsimonious models performed slightly lower than the QIC-driven models but by no more than 0.01 AUC point (S3 Table).

Considering that claims more distant from the index date potentially offer greater lead time, we developed a prediction model based on claims 16–24 months prior to PDAC diagnosis, for which the AUC (0.552) was lower than that of the <15 months model. (S3 Table).

## Discussion

In this analysis of older adults in the U.S., we showed that healthcare claims for risk factors and PDAC-related symptoms and signs start to increase months ahead of PDAC diagnosis and that healthcare utilization intensifies nearing the time of PDAC diagnosis. The AUC of the prediction model built on 15 months of claims prior to the index date reached 0.68 when all study subjects were considered and 0.73 among persons with new-onset diabetes. With omission of claims in the three months before diagnosis, the AUCs dropped substantially both for all cases and controls (0.58) and for persons with new-onset diabetics (0.63). At a specificity threshold of 99%, models that incorporate all claims with 15 months of index date have limited sensitivity of 16–18%, which drops to 4–5% by excluding the final 3 months of claims.

Two previously published models have focused on new-onset diabetes: one a model based on new-onset U.K. diabetes patients aged ≥50 years that incorporated clinical diagnosis as well as laboratory data from electronic health records, [12] another a model based on biochemically-determined new-onset diabetes patients aged ≥50 years in Olmsted County, Minnesota, that incorporated data on changes in glucose and weight. [13] The U.K. model reached an AUC of 0.82 by internal validation and the Olmsted County model reached an AUC of 0.87 by external validation within another population in Olmsted county (S4 Table). Our model in new-onset diabetes patients, with AUC of 0.73, differs from previous models on three major aspects: age range, regional scope, and type of data. Our study population comprised persons aged ≥ 68 years, who have higher baseline incidence of type 2 diabetes than younger persons, therefore the likelihood that a recent diagnosis of diabetes could be attributable to pancreatic cancer is lower. Our model comprised Medicare patients spanning 28 SEER regions in the nation. Variability in documenting and billing clinical diagnoses may have been greater than in the U.K. and in Olmsted County, with health systems that are less heterogeneous. [32, 33] Finally, our model relied on insurance claims, rather than medical records, thus information on laboratory test results and self-reported complaints were lacking. Because continuous formats of laboratory test results (e.g., glucose level) and weight provide more granular information on physiological state than binary diagnoses, incorporating such parameters may explains more of the variation in PDAC risk.

Previously published pancreatic cancer prediction models on populations not selected by diabetes status include a Korean nationwide study that incorporated laboratory data from regular health examinations (AUC = 0.81), [34] a population-based case-control study in Connecticut incorporating questionnaire-based data on ethnic ancestry, ABO blood group, smoking cessation, pancreatitis and recent use of proton-pump inhibitor medications (AUC = 0.764), [11] and a pancreatic cancer consortium (PanScan) analysis of multiple observational studies with questionnaire-based data on epidemiologic risk factors and blood group genotype (AUC = 0.61). [35] (S4 Table) Our prediction model in the overall population reached AUC of 0.68, which was lower compared to that estimated in the Korean and Connecticut models. We attribute lower performance to the lack of information on laboratory test results and medications, to the lack of self-reported data not available in claims databases, as well as to the older age of our population (≥68 year). In addition to the advantage of laboratory tests described above, over-the-counter medications like proton-pump inhibitors provide indications of abdominal pain prior to seeking help from health professional, thus adding more granular and potentially earlier information on subclinical health changes. Also, Medicare claims data do not include lifestyle risk factors of PDAC, such as smoking and alcohol consumption, and family history of cancer, which increase the risk of PDAC. [36–38] The availability of such risk factor data would have improved our models. Considering that models incorporating data on health changes leading up to pancreatic cancer diagnosis performed better than the PanScan model that relied on data on static etiologic risk factors and ABO genotypes [35] suggests that models based on such etiologic risk factors do not well identify exactly when such factors should operate, compared to prediction models based on changes in health.

Whether prediction models based on recent changes in health aid in detecting cancer sufficiently early enough for better treatment options, especially potentially curative resection or aggressive multifractionated radiation, is a critical question. Our sensitivity analysis results excluding the final 3 months of claims before index date led to a substantial drop in AUC. One of the strongest predictors was jaundice, which was associated with 24-fold risk of PDAC including all claims within 15 months of index date and 3.8-fold risk excluding the final 3 months. The odds ratios for other strong predictors of pancreatic cancer, such as chronic pancreatitis, acute pancreatitis, abdominal pain and weight loss also attenuated substantially when the final 3 months of claims were excluded. With longitudinal data from healthcare claims, we observe that healthcare claims are comparatively more present in PDAC patients than controls prior to PDAC diagnosis; however, often these health changes are noted very close (<3 months) to the diagnosis of PDAC, thus limiting their predictive value for early detection.

In our analyses, one limitation of using Medicare files is that healthcare claims not billed to Medicare would not have been reflected in the files. By restricting the population to those continuously enrolled in both Medicare Parts A (inpatient care) and B (outpatient care), we limited the population to those who have opted for fee-for-service outpatient reimbursement through Medicare, which therefore would have records of most services covered for its members. Another limitation of Medicare claims data is that claims do not distinguish incident from prevalent conditions. Indeed, knowing the duration of a condition since onset can help improve the model as demonstrated by Risch et al. [11] For conditions like diabetes, pancreatitis and dyspepsia, the strength of the association with PDAC decreases with time since onset; thus, parameterizing the timing of the onset of disease would enhance the fit of the model. Another limitation of Medicare claims data is the lack of representation of younger people who may still be at risk of PDAC. Regardless, the mean age of PDAC diagnosis is 70, [17] thus our model applies to a majority of older persons in U.S at risk for PDAC. Although we aimed to include a comprehensive list of risk factors and symptoms of PDAC, some factors may not have been represented in our analysis. An example is back pain, which has been associated PDAC with odds ratios ranging from 1.3 to 1.4. [39, 40] While including additional factors could improve the prediction model, relatively weak associations are unlikely to improve the predictive performance of the model appreciably.

## Conclusion

We created a PDAC prediction model that applies to Medicare enrollees living in SEER regions in the U.S. The model provides some information bearing upon the emergent diagnosis of pancreatic cancer, but not enough on its own to be useful in population screening. Excluding the final 3 months of claims prior to PDAC diagnosis reduced the discriminative performance of the model appreciably. Future models should consider sensitivity analyses excluding health changes noted in the final months of PDAC diagnosis in order to evaluate true clinical utility of prediction models for PDAC early detection.

## Supporting information

S1 Table Covariates of pancreatic cancer and their ICD-9 codes. (DOCX)

S2 Table Ratio of % of cases to controls with a healthcare claim for a covariate within a 24-month period prior to pancreatic cancer diagnosis, by 3-month interval. (DOCX)

S3 Table Summary of performance measures on QIC-drive multivariable models and parsimonious models. (DOCX)

S4 Table Summary of previous studies on prediction modeling of pancreatic cancer and current model. (DOCX)
