# Supplementary material for: Do changes in health reveal the possibility of undiagnosed pancreatic cancer? Development of a risk-prediction model based on healthcare claims data
Source: PLoS One. 2019 Jun 25;14(6):e0218580. doi: 10.1371/journal.pone.0218580 (PMC6592596; doi:10.1371/journal.pone.0218580)
Supplement: S1 Table — (DOCX) [file pone.0218580.s001.docx]

**S1 Table**

**Indicators of Pancreatic Cancer and Their ICD-9 Codes**

| **Variables** | **ICD-9** |
| --- | --- |
| ***Clinical Diagnoses*** |  |
| Acute pancreatitis | 577.0 |
| Chronic pancreatitis | 577.1 |
| Diabetes | 250.xx, excluding type I (250.x1, 250.x3) |
| Poorly controlled DM | 250.xx, excluding 250.00 and type I (250.x1, 250.x3) |
| Dyspepsia, Gastritis, Peptic ulcer disease | 536.8, 535.5, 533.90, 533.91 533.00-533.61 |
| Gallbladder Disease | 575.9 |
| Acute cholecystitis | 575.0 |
| Depression | 311, 2962, 2963, 2965, 2966, 2967, 2980, 30110, 30113, 3090, 3091 |
| ***Symptoms and Signs*** |  |
| Abdominal Pain | 789.0-789.09 |
| Upper Abdominal pain (right, left, epigastric) | 789.01, 789.02, 789.06 |
| Chest Pain | 786.50-786.52, 786.59, 413.9 |
| Gastrointestinal symptoms |  |
| Abnormal feces | 787.7 |
| Other symptoms involving digestive system | 787.99 |
| Flatulence, eructation, and gas pain | 787.3 |
| Change in bowel habits | 787.9 |
| Constipation (atonic, simple, spastic) | 564.0 |
| Diarrhea | 787.91 |
| Irritable bowel | 536.9 |
| Esophageal reflux | 530.81 |
| Jaundice | 782.4 |
| Weight loss/Anorexia/Cachexia |  |
| Anorexia | 783.0 |
| Abnormal loss of weight and underweight | 783.21, 783.22 |
| Cachexia | 799.4 |
| Feeding difficulties and mismanagement | 783.3 |
| Nausea and Vomiting |  |
| Nausea with vomiting | 787.01 |
| Nausea alone | 787.02 |
| Vomiting alone | 787.03 |
| Malaise/fatigue | 780.7 |
| Itching/pruritis | 698.9 |
