# Supplementary material for: Do changes in health reveal the possibility of undiagnosed pancreatic cancer? Development of a risk-prediction model based on healthcare claims data
Source: PLoS One. 2019 Jun 25;14(6):e0218580. doi: 10.1371/journal.pone.0218580 (PMC6592596; doi:10.1371/journal.pone.0218580)
Supplement: S2 Table — (DOCX) [file pone.0218580.s002.docx]

**S2 Table**

**Ratio of % of cases to controls with a healthcare claim for a clinical indicator within a 24 month period prior to pancreatic cancer diagnosis, by 3 month interval**

|  | Q8 | Q7 | Q6 | Q5 | Q4 | Q3 | Q2 | Q1 |
| --- | --- | --- | --- | --- | --- | --- | --- | --- |
| Acute pancreatitis | 2.8 | 3.8 | 3.7 | 4.0 | 4.7 | 7.4 | 8.1 | 36.3 |
| Chronic pancreatitis | 3.2 | 4.8 | 4.0 | 6.3 | 8.3 | 10.5 | 16.5 | 39.8 |
| Diabetes mellitus | 1.4 | 1.4 | 1.4 | 1.5 | 1.5 | 1.6 | 1.6 | 1.9 |
| Dyspepsia, gastritis, peptic ulcer disease | 1.2 | 1.2 | 1.1 | 1.1 | 1.2 | 1.4 | 1.7 | 4.5 |
| Gallbladder disease | 1.3 | 0.7 | 1.3 | 1.7 | 1.5 | 2.0 | 3.7 | 18.0 |
| Acute cholecystitis | 2.0 | 1.2 | 0.7 | 1.5 | 1.6 | 2.2 | 3.0 | 11.0 |
| Depression | 0.9 | 0.9 | 0.8 | 0.8 | 0.9 | 1.0 | 1.0 | 1.3 |
| Any abdominal pain | 1.2 | 1.2 | 1.1 | 1.2 | 1.3 | 1.6 | 2.0 | 6.6 |
| Chest pain | 1.2 | 1.1 | 1.1 | 1.1 | 1.2 | 1.2 | 1.3 | 2.0 |
| Gastrointestinal symptoms | 1.1 | 1.1 | 1.0 | 1.0 | 1.2 | 1.4 | 1.6 | 3.7 |
| Esophageal reflux | 1.0 | 1.0 | 1.0 | 1.0 | 1.1 | 1.1 | 1.2 | 2.2 |
| Jaundice | 2.0 | 4.0 | 4.0 | 3.4 | 5.8 | 6.8 | 10.5 | 140.8 |
| Weight loss / Anorexia / Cachexia | 1.0 | 1.2 | 1.3 | 1.4 | 1.3 | 2.0 | 2.7 | 8.6 |
| Nausea and/or vomiting | 1.1 | 1.1 | 1.0 | 1.0 | 1.2 | 1.3 | 1.4 | 4.5 |
| Malaise/Fatigue | 0.8 | 0.7 | 1.0 | 1.3 | 1.0 | 1.5 | 1.5 | NA |
| Itching/pruritic | 0.9 | 1.1 | 1.0 | 1.1 | 1.1 | 1.3 | 1.1 | 3.8 |
