# Supplementary material for: Do changes in health reveal the possibility of undiagnosed pancreatic cancer? Development of a risk-prediction model based on healthcare claims data
Source: PLoS One. 2019 Jun 25;14(6):e0218580. doi: 10.1371/journal.pone.0218580 (PMC6592596; doi:10.1371/journal.pone.0218580)
Supplement: S3 Table — (DOCX) [file pone.0218580.s003.docx]

**S3 Table**

**Summary of Performance Measures on QIC-driven Multivariable Models and Parsimonious Models and Sensitivity Analyses**

| **Time period for claims included in the multivariable model** | **Performance measure** | **Model** | |
| --- | --- | --- | --- |
|  |  | **Best multivariable model per QIC** | **Parsimonious model** * |
| 1-15 month prior to index date | AUC (95% CI) | 0.683 (0.680-0.687) | 0.672 (0.668-0.676) |
|  | Optimism-corrected AUC (95% CI) | 0.682 (0.678-0.686) | 0.671 (0.667-0.675) |
| 4-15 month prior to index date | AUC (95% CI) | 0.578 (0.575-0.582) | 0.576 (0.572-0.580) |
|  | Optimism-corrected AUC (95% CI) | 0.577 (0.573-0.580) | 0.574 (0.570-0.578) |
| 16-24 month prior to index date | AUC (95% CI) | 0.552 (0.548-0.555) | 0.550 (0.546-0.554) |
|  | Optimism-corrected AUC (95% CI) | 0.549 (0.545-0.553) | 0.548 (0.544-0.552) |
| New-onset DM: 1-15 month prior to index date | AUC (95% CI) | 0.735 (0.721-0.748) | 0.732 (0.718-0.745) |
|  | Optimism-corrected AUC (95% CI) | 0.730 (0.717-0.744) | 0.727 (0.713-0.741) |
| New-onset DM: 4-15 month prior to index date | AUC (95% CI) | 0.635 (0.621-0.650) | 0.627 (0.612-0.641) |
|  | Optimism-corrected AUC (95% CI) | 0.626 (0.612-0.641) | 0.619 (0.604-0.634) |
| New-onset DM: 16-24 month prior to index date | AUC (95% CI) | 0.567 (0.552-0.582) | 0.554 (0.539-0.569) |
|  | Optimism-corrected AUC (95% CI) | 0.551 (0.536-0.566) | 0.540 (0.525-0.555) |

* Parsimonious model includes matching factors (sex, age at diagnosis, and year of diagnosis), race, acute pancreatitis, chronic pancreatitis, diabetes, abdominal pain, weight loss and jaundice.
