# Supplementary material for: Do changes in health reveal the possibility of undiagnosed pancreatic cancer? Development of a risk-prediction model based on healthcare claims data
Source: PLoS One. 2019 Jun 25;14(6):e0218580. doi: 10.1371/journal.pone.0218580 (PMC6592596; doi:10.1371/journal.pone.0218580)
Supplement: S4 Table — (DOCX) [file pone.0218580.s004.docx]

**S4 Table**

**Summary of Previous Studies on Prediction Modeling of Pancreatic Cancer and Current Model**

| **Author and publication year of published model** | **Type of data** | **Study population** | **Generalizable population** | **Study design** | **Timeframe of data collection relative to PDAC diagnosis** | **Validation** | **Performance measure** | **Variables in the final model** |
| --- | --- | --- | --- | --- | --- | --- | --- | --- |
| ***New-Onset Diabetes*** | | | | | | | | |
| Current model | Medicare claims data | New-Onset Diabetes in PDAC cases and controls among Medicare enrollees | U.S. Medicare population | Case-control | Prospective | Internal validation | AUC = 0.73 | Age, sex, race, influenza vaccine, acute and chronic pancreatitis diabetes, dyspepsia, depression, abdominal pain, weight loss, jaundice, nausea/vomiting |
| Sharma et al. 2018 | Electronic health records | New-Onset Diabetes in Rochester Epidemiology Cohort in Olmsted County | Olmsted County | Cohort | Prospective | External validation within Olmsted County | AUC = 0.87 | Age, change in weight, change in blood glucose |
| Boursi et al. 2017 | Electronic health records | U.K. Residents with new-onset diabetes defined by diagnostic codes | U.K. residents who are seen by general practitioners | Cohort | Prospective | Internal validation | AUC = 0.82 | Age, BMI, change in BMI, smoking, insulin, metformin, PPI, A1C, hemoglobin, creatinine, ALP, cholesterol |
| ***General population, unselected by diabetes status*** | | | | | | | | |
| Current model | Medicare claims data | PDAC cases and controls among Medicare enrollees | U.S. Medicare population | Case-control | Prospective | Internal validation | AUC = 0.68 | Age, sex, race, influenza vaccine, acute and chronic pancreatitis, depression, abdominal pain, chest pain, weight loss, jaundice |
| Yu et al. 2016 | Health examination data | Working Koreans and dependents enrolled in the Korean National Health Insurance Corporation (NHIC) | Employed population and their dependents in Korea | Cohort | Prospective | External validation within NHIC AUC = 0.81 | AUC = 0.81 in men, 0.80 in women | Age, Sex, Height, BMI, Smoking, alcohol, Blood and urine glucose |
| Risch et al. 2016 | Questionnaire data | PDAC Cases and controls in Connecticut | Residents of Connecticut | Case-control | Retrospective | None | AUC = 0.76 | Age, sex, race, Jewish ancestry, Smoking and cessation, family history of pancreatic cancer; diabetes and duration; pancreatitis, PPI, H. pylori, non-AB blood group |
| Klein et al. 2013 | Questionnaire data and ABO genotypes | PanScan case-control or cohort study participants (12 studies) | PanScan study sites | Case-control and cohort | Retrospective and prospective | None | AUC = 0.61 | Age, sex, BMI, Smoking, alcohol, diabetes (>3 years duration); family history of pancreatic cancer, ABO genotype; SNPs in the ABO genes |
